# Conspiracy Mentality Predicts Public Opposition to Foreign Trade

**DOI:** 10.3389/fpsyg.2021.658919

**Published:** 2021-06-17

**Authors:** Alexander Jedinger

**Affiliations:** GESIS – Leibniz Institute for the Social Sciences, Cologne, Germany

**Keywords:** conspiracy mentality, policy attitudes, foreign trade, protectionism, economic globalization

## Abstract

The proliferation of protectionist sentiments and policies has raised questions about the psychological sources of trade openness among the public. The current research investigated the effects of a previously neglected factor on attitudes toward international trade: conspiracy mentality. Conspiracy mentality describes the generalized belief that political and economic events are controlled by powerful malevolent forces acting in secret. Using data from a cross-sectional survey of German adults (*N* = 391), I hypothesized and found that conspiracy mentality is uniquely associated with the perceived threat posed by foreign trade and opposition to international trade. These findings suggest that individual differences in conspiracy mentality make an important contribution to understanding the fears associated with economic globalization.

## Introduction

In recent years, there has been heated public debate about the political, social, and economic consequences of international trade (Blendon et al., 2017). Although general support for foreign trade remains high in most Western industrialized nations, free trade agreements such as the Transatlantic Trade and Investment Partnership (TTIP) have witnessed growing skepticism among the public (Bluth, 2016; White, 2017; Steiner, 2018; Eliasson and Huet, 2019). In addition, many countries adopted protectionist policies after the global financial crisis to shield their domestic economy against foreign imports (Evenett and Fritz, 2019; World Trade Organization, 2019). This development recently peaked under former U.S. President Donald Trump when he withdrew the United States from key international trade agreements (e.g., the Trans-Pacific Partnership; TPP) and enacted several increases in tariffs and other trade barriers that culminated in an ongoing trade conflict with Europe and China (Chong and Li, 2019; Fajgelbaum et al., 2020). Against this background, understanding the psychological determinants of trade openness has become an important task for scholars from various disciplines because protectionist attitudes have been linked to rising levels of support for right- and left-wing populist candidates and parties that use anti-globalization rhetoric to rally against immigrants and financial elites (Rodrik, 2018; van der Waal and de Koster, 2018; van Bohemen et al., 2019).

Protectionist attitudes entail support for policies that restrict international trade to protect the domestic economy from foreign competition (e.g., import tariffs or non-tariff trade barriers; see Rodrik, 1995). Previous research has largely attributed protectionist sentiments to economic insecurity among low-skilled individuals in export-oriented industries who feel threatened by foreign competition (e.g., O'Rourke and Sinnott, 2001; Scheve and Slaughter, 2001; Kaltenthaler et al., 2004; Mayda and Rodrik, 2005). Notably, however, the material advantages and disadvantages of global trade are of little importance in predicting attitudes toward foreign trade (e.g., Johnston, 2013; Rho and Tomz, 2017). Over and above material self-interest, recent work has argued that views on global trade are driven by generalized political attitudes, including nationalism (O'Rourke and Sinnott, 2001; Rankin, 2001), ethnocentrism (Mansfield and Mutz, 2009; Mansfield et al., 2019), authoritarianism and social dominance orientation (Johnston, 2013; Satherley and Sibley, 2016; Mutz and Kim, 2017; Jedinger and Burger, 2020). In the present study, I contribute to the growing research on the psychological basis of trade attitudes by exploring the potential role of conspiracy mentality in understanding the psychological differences between supporters and opponents of free trade.

## Conspiracy Mentality and Attitudes Toward Foreign Trade

Conspiracy theories have long been a pervasive feature of societal discourses, especially in times of social and economic crises (van Prooijen and Douglas, 2017). Conspiracy theories are defined as attempts to explain important societal events by the planned actions of sinister and powerful forces, which act in the hidden (van Prooijen, 2018; Douglas et al., 2019). For example, during the Great Recession of 2008/2009, a popular conspiracy theory proposed that the financial crisis was deliberately caused by Wall Street bankers to expand the power of the American Federal Reserve System (Oliver and Wood, 2014).

Empirical research consistently finds that beliefs in specific kinds of conspiracy theories are strongly intercorrelated, which suggests an underlying predisposition for conspiracy explanations (e.g., Goertzel, 1994). This is true for conspiracy theories that are relatively independent, fictitious, or even contradictory (Swami et al., 2011; Wood et al., 2012; Brotherton et al., 2013). Accordingly, several scholars have proposed the existence of a conspiratorial mindset or conspiracy mentality. Conspiracy mentality refers to a general propensity to view societal events as the result of the secret plans of powerful individuals and groups that pursue their own unlawful or malicious goals (Moscovici, 1987; Brotherton et al., 2013; Imhoff and Bruder, 2014).

During the last decade, scholars have begun to systematically investigate the real-world consequences of conspiracy beliefs, covering a wide range of issues from the environment to public health and immigration, just to name a few (for an overview, see Douglas et al., 2015). However, there has been a scarcity of research that explored how endorsements of conspiracy narratives are related to economic attitudes and behavior.

The idea that a clandestine group of powerful people controls important economic and financial events is very popular, and a malicious plot to rig the economy is part of many conspiracy narratives (Uscinski, 2020). For instance, a common theme within the so-called New World Order conspiracy is that a global elite who secretly controls international organizations such as the World Bank, the International Monetary Fund (IMF), and the World Trade Organization (WTO) is pursuing the worldwide dismantling of trade barriers to establish an authoritarian world government (Spark, 2000).

Economic phenomena such as globalization and international trade provide fertile ground for conspiratorial thinking because rapid economic changes elicit feelings of material insecurity such as those associated with technological progress, offshoring, and automation (van Prooijen and Douglas, 2017). Additionally, economic issues are often complex and difficult for citizens to comprehend, which in turn attracts people to simplistic explanations to gain a sense of control (Baron and Kemp, 2004; Leiser et al., 2017). Finally, public distrust in key actors of the free market economy, such as major corporations and banks, is widespread and fuels conspiratorial suspicion (Smallpage et al., 2017; Gallup, 2020).

The uncertainties and complexity surrounding foreign trade raise the possibility that conspiracy mentality may be an important and previously overlooked explanatory factor for protectionist attitudes. According to Imhoff and Bruder (2014), conspiracy mentality is a belief system that entails a heightened sensitivity to cues that signal power asymmetries in intergroup relations. More specifically, the extent to which people subscribe to a conspiracy mentality is associated with hostility toward powerful groups that are held responsible for undesirable developments in society. The concept of conspiracy mentality can be situated within a larger theoretical framework of ideology as motivated social cognition (Jost et al., 2003). Jost et al. (2003) identified two core elements underlying ideological belief systems: advocating vs. resisting social change and opposing vs. accepting social inequality. These core elements are linked to basic epistemic, existential and relational needs. In other words, all individuals fundamentally strive for epistemic certainty, physical security and positive feelings associated with belonging to important social in-groups. The theory posits that those higher in need of managing uncertainty and threats are more likely to adapt conservative system-justifying belief systems because they provide a functional match to their basic psychological needs.

Resistance to change is most often measured by right-wing authoritarianism (Altemeyer, 1996), which predicts negative attitudes toward groups perceived as culturally deviant and threatening to the norms and values of in-groups (Shaffer and Duckitt, 2013). Acceptance of inequality is often related to individual differences in social dominance orientation (Pratto et al., 1994) that give rise to negative attitudes toward groups of low socioeconomic status (Asbrock et al., 2010). In contrast, conspiracy mentality has been conceived as a system-challenging belief system because it entails an aversion toward those in power (Imhoff and Bruder, 2014). However, recent work argued that a conspiratorial worldview is a means to bolster the societal status quo by attributing negative developments in society to small groups of powerful but evil-minded conspirators rather than to deficits of the social system as a whole (Douglas and Sutton, 2018). Consistent with this reasoning, several studies have indicated that threats to the legitimacy of the social system actually lead to greater endorsements of conspiracy beliefs (Federico et al., 2018; Jolley et al., 2018).

Conspiracy mentality also shares some conceptual similarities with populist attitudes. Populism is defined as a generalized attitude in which society is perceived as an antagonism between the “pure people” and a corrupt political elite (Mudde, 2004). The elite is assumed to act only for its material benefit and against the interests of the people, while the people are assumed to have higher moral qualities. The skepticism toward power elites and the Manichean view of the world are shared by people high in conspiracy mentality and populist attitudes (Castanho Silva et al., 2017). However, conspiracy beliefs aim more at reducing epistemic uncertainty and the associated loss of control by offering explanations for specific events that differ from official accounts (Swami et al., 2010; Imhoff and Bruder, 2014). Furthermore, populist beliefs are primarily focused on the role of the political establishment, whose actions are not necessarily conspiratorial. Finally, an essential component of populism is the alignment of political decision-making with the “will of the people.” People with a pronounced conspiracy mentality, on the other hand, see themselves as superior to their fellow citizens because they feel “enlightened” and have seen through the supposed conspiratorial machinations (Imhoff and Lamberty, 2017). Thus, although conspiracy mentality and populist attitudes have conceptual similarities, they indicate different patterns of generalized attitudes.

Another line of inquiry focuses more on the consequences of a conspiratorial mindset. Past research has shown that people high in conspiracy mentality are more likely to express prejudice toward powerful outgroups (e.g., managers and capitalists), distrust the government, and tend to attribute less credibility to experts (Imhoff and Bruder, 2014; Einstein and Glick, 2015; Imhoff and Lamberty, 2018; Imhoff et al., 2018). Furthermore, they are less likely to engage in conventional forms of political participation (e.g., voting), which are deemed ineffective in challenging existing political and economic power structures; however, they are more prone to rely on alternative forms of political participation, such as violent protests (Jolley and Douglas, 2014; Uscinski et al., 2016; Ardèvol-Abreu et al., 2020; Imhoff et al., 2021).

More directly relevant for the current investigation, past research suggests that people who endorse a conspiratorial worldview are more likely to oppose policies promoted by powerful agents (e.g., major corporations or national governments) because they are supposed of serving the sinister interests of clandestine elites (Imhoff et al., 2018; Lamberty and Imhoff, 2018). For instance, Lamberty and Imhoff (2018) demonstrated that individuals high in conspiracy mentality evaluated a fictitious drug more positively if its approval was advocated by a low-power group (an interest group of patients) than if the drug was supported by a high-power group (a pharmaceutical consortium).

In the context of the current investigation, trade negotiations often involve asymmetrical power relations between citizens, on the one hand, and major corporations, international financial institutions, and national authorities, on the other hand. Consequently, the social, economic, and cultural consequences of foreign trade will be perceived as a threat to the well-being of citizens among those higher in conspiracy mentality. Furthermore, any policy that promotes the dismantling of trade restrictions will be seen as a malicious plot to the detriment of the interests of citizens and therefore opposed by those high in this predisposition.

## The Present Research

Although previous research has examined the impact of generalized political attitudes on protectionist sentiments, we know relatively little about how a conspiratorial worldview predicts aversion to economic openness. To address this gap, the current study examines the extent to which conspiracy mentality affects public opposition to free trade using data from a cross-sectional survey among German Internet users. According to the theoretical framework presented above, individuals high in conspiracy mentality will perceive policies proposed by high-power agents as suspicious. Free trade agreements and the dismantling of regulatory barriers to trade have long been promoted by the German government and influential business associations, as the German export-oriented economy is highly integrated into global trade (Federal Ministry for Economic Affairs Energy, 2020). Thus, I hypothesize that conspiracy mentality will be positively associated with the endorsement of protectionist attitudes (Hypothesis 1) and should increase perceptions of the economic, social, and cultural threat posed by international trade (Hypothesis 2).

I also control for several established covariates that are related to trade preferences, such as resistance to change, acceptance of social inequality, labor market skills, demographics, as well as populist attitudes.

## Method

### Participants and Procedure

The sample for the present study is a subsample (*N* = 391) of a larger survey (*N* = 1, 000) on the consequences of conspiracy beliefs among German citizens. A national quota sample of adult German citizens was drawn from an opt-in online panel maintained by a commercial survey agency (Respondi). The quotas were set up to represent the German population with internet access in terms of age, gender, education, and region of residence. The survey was administered online between March 9 and 16, 2020. The participants received a small financial reward from the survey agency in exchange for their participation. In total, *N* = 1,000 participants completed the survey, and a randomly chosen subsample of *N* = 500 answered questions about their trade policy preferences. Of these participants, 109 were excluded after listwise deletion of missing data^1^. A sensitivity power analysis (α = 0.05, two-tailed) indicated that a sample size of 391 allows the detection of a relatively small effect (*f* ^2^ = 0.02) with 80% power. The age of the remaining participants varied between 19 and 76 years (*M* = 50.4, *SD* = 15.1), and about half of the participants were male (51.7%). Regarding their formal level of education, 20.7% reported having a lower secondary qualification (after 9 years of schooling) or no degree, 43.5% reported having an intermediary secondary qualification (after 10 years of schooling), and 35.8% reported having a higher secondary qualification (technical college or University entrance qualification, after 11 or 12 years of schooling). The median monthly household income category was 2,500 to <3,000 euros. The distribution of age and gender in the sample largely corresponds to the German population, even after respondents with missing data were excluded. Participants with a lower education level were underrepresented, while participants with a medium level of education were slightly overrepresented (a detailed comparison can be found in Supplementary Table 2.1).

After providing informed consent, the participants completed a questionnaire including measures of economic attitudes, conspiracy beliefs, political attitudes, and a host of other items unrelated to the present research. The exact wording of all measures can be found in Supplementary Material, Section 1. Ethical approval was obtained from the local institutional research and ethics committee.

### Measures

#### Conspiracy Mentality

Conspiracy mentality was measured with a shortened five-item version of the original Conspiracy Mentality Scale proposed by Imhoff and Bruder (2014). All items were presented in randomized order and scored on five-point scales ranging from 1 (*strongly disagree*) to 5 (*strongly agree*). The descriptive statistics and corrected item-total correlations of the items are shown in Table 1. The items were averaged to produce a composite score (Cronbach's α = 0.82; McDonald's ω = 0.84).

**Table 1:** Descriptive item statistics for the conspiracy mentality scale.

**Items**		***M***	***SD***	**r_**it**_**
1.	Most people do not recognize the extent to which our lives are determined by conspiracies that are concocted in secret.	2.77	1.32	0.72
2.	There are secret organizations that have great influence on political decisions.	3.13	1.28	0.73
3.	Politicians and other leaders are nothing but the puppets of powers operating in the background.	3.30	1.21	0.70
4.	I think that the various conspiracy theories circulating in the media are absolute nonsense.^*^	3.52	1.17	0.50
5.	There is no good reason to distrust governments, intelligence agencies, or the media.^*^	2.47	1.14	0.41

The entries are the means, standard deviations, and corrected item-total correlations. The items marked with an asterisk ^*^ are reverse coded. Response scale: 1, strongly disagree, 2, disagree, 3, neither agree nor disagree, 4, agree, and 5, strongly agree. N = min. 367.

#### Attitudes Toward International Trade

General opposition to international trade was measured by one item “(What do you think about the growing trade relations between Germany and other countries—do you think it is a good thing, or a bad thing for Germany?”) adapted from Bluth (2016). The participants were asked to rate their attitude toward growing trade ties with other countries on a seven-point scale (1 = *very bad*; 7 = *very good*). Furthermore, they were asked to indicate their support for free trade agreements between the European Union (EU) and other countries using a self-developed item (“Currently, there is much discussion about the adoption of free trade agreements. Do you favor or oppose that the European Union (EU) enters free trade agreements with other countries?”). Responses were given on a seven-point scale (1 = *completely support*; 7 = *completely oppose*).

#### Perceived Threats Posed by International Trade

To assess perceived threats associated with international trade, the participants were asked to indicate whether growing global trade had positive or negative consequences for Germany in 10 different areas using items adapted from Bluth (2016): economic growth; employment and the labor market situation; the consumer prices of goods and services; international competitiveness; consumer protection standards (e.g., for agricultural products); environmental standards; workers' rights and social standards; cultural life; state regulatory jurisdiction; and cultural values and traditions. These areas were selected because they constitute the main lines of public dissent on the anticipated positive and negative consequences of international trade. The participants responded to these items, which were presented in a randomized order, on scales ranging from 1 (*very positive*) to 7 (*very negative*).

To examine the factorial structure of the 10-item scale, a principal axis factor analysis was conducted. The initial eigenvalues and visual inspection of the scree plot suggested that a single factor should be extracted. The first unrotated factor (eigenvalue = 5.24) accounted for 94.3% of the total variance, with factor loadings ranging between 0.66 and 0.76. A parallel analysis, according to Horn (1965), suggests the extraction of three factors. After oblique rotation (promax), which takes into account that the factors are correlated, the three-factorial solution could be clearly interpreted. The first factor describes the perceived economic threats posed by foreign trade (four items). The second factor refers to the undermining of environmental, social, and product-related standards (four items). The third factor refers to the negative consequences of foreign trade for cultural values (two items). All factors were strongly correlated (*r* ≥ 0.57). The following results, however, do not differ substantively by specific facets of trade threats (see Supplementary Materials, Supplementary Table 3-1)^2^. Therefore, I use a composite scale of perceived threat in the remaining analyses (Cronbach's α = 0.91; McDonald's ω = 0.92).

#### Resistance to Change and Acceptance of Inequality

Generalized political attitudes indicating a tendency to derogate out-groups and favor in-groups emerged as robust predictors of trade attitudes, often rivaling the effects of more tangible consequences of foreign trade (e.g., Mansfield and Mutz, 2009; Johnston, 2013). According to Jost et al. (2003), generalized political attitudes are based on individual differences in the propensity to tolerate social changes and hierarchies. Resistance to social change was captured by two items adapted from the Moral Traditionalism Scale (Conover and Feldman, 1986). The items include “The world is constantly changing, and we should adapt our understanding of moral behavior to these changes” and “We should be more tolerant of people who choose to live according to their own moral standards, even if they are very different from our own” (both are reverse coded). Acceptance of social inequality was measured with three items taken from the Social Inequality Scale (Mayer et al., 2014) with statements such as “The differences in rank between people are acceptable because they essentially express what you did with the opportunities you had.” Participants responded on five-point Likert scales (1 = *strongly disagree*; 5 = *strongly agree*), and an average score was computed for resistance to change (Cronbach's α = 0.45; McDonald's ω = 0.67) and acceptance of inequality (Cronbach's α = 0.68; McDonald's ω = 0.68).

#### Populism

Populism is a generalized attitude that divides society into deceitful elites and the “true people” and demands that political decision-making should be the expression of a homogeneous “popular will” (Mudde, 2004). While populist attitudes are essentially content-free because they refer to the inherent dualism between political elites and ordinary people, the anti-elitist component and distrust in political authorities could possibly influence attitudes toward foreign trade. Therefore, populist attitudes are included as a covariate in the following analyses. Participant completed a six item populism scale (Akkerman et al., 2014) that included statement such as “The political differences between the elite and the people are larger than the differences among the people” (1 = *strongly disagree*; 5 = *strongly agree*). The scale exhibited very good reliability (Cronbach's α = 0.86; McDonald's ω = 0.87).

#### Labor Market Skills

According to classical trade theory, individuals with higher levels of labor market skills and those working in export-oriented sectors of employment are more supportive of trade openness (Rodrik, 1995). Unfortunately, the survey does not include questions about the respondents' occupational industry. However, I will use educational attainment, task autonomy, and income as established indicators of individuals' objective skill level (Scheve and Slaughter, 2001; Blonigen, 2011; Blonigen and McGrew, 2014). First, the participants were asked to report their level of formal education and vocational qualifications, which were combined to produce an overall measure of educational attainment, as outlined by Hoffmeyer-Zlotnik and Warner (2005) (1 = *no professional qualification*; 5 = *University degree*). Second, the participants indicated their occupational category (manual laborer, clerk, civil servant, self-employed worker, or farmer), followed by their level of professional activity (e.g., frontline or managerial employee). The professional activity was then categorized based on the degree of task autonomy (Hoffmeyer-Zlotnik and Geis, 2003), with higher values indicating increasing responsibility and leadership skills and lower task routineness, resulting in a five-point scale ranging from 1 (*low autonomy*) to 5 (*high autonomy*). Finally, the participants reported their monthly household income using 12 categories that were recoded to income quintiles (1 = < *1.500 euro*; 5 = *4,000 euro or more*).

#### Demographics

The following demographic controls were included: age (in years), gender (1 = *male*, 0 = *female*), and region (1 = *East Germany*, 0 = *West Germany*).

## Results

The descriptive statistics and bivariate correlations among the main study variables are displayed in Table 2. Consistent with the hypotheses formulated above, conspiracy mentality is positively correlated with opposition to free trade in general and free trade agreements between the EU and other states. Furthermore, a conspiratorial worldview is positively correlated with increased levels of perceived threat posed by international trade. In turn, perceived threat strongly correlates with opposition to trade openness and trade agreements. Resistance to social change was positively related to trade protectionism in general and perceived trade threats but unrelated to attitudes toward trade agreements. Conversely, the acceptance of social hierarchies was negatively associated with the disapproval of trade negotiations and beliefs about trade threats but unrelated to general protectionism. As expected, the correlation analysis revealed significant positive associations between populism and all three indicators of protectionism. There was also a strong positive correlation between populist attitudes and conspiracy mentality. In line with previous research, individuals with higher levels of educational attainment, task autonomy and income are less likely to oppose trade openness, but only individuals who score higher on task autonomy endorse the negotiation of free trade agreements.

**Table 2:** Descriptive statistics and correlations among the main study variables.

	***M***	***SD***	**1**	**2**	**3**	**4**	**5**	**6**	**7**	**8**	**9**
1. Conspiracy mentality	3.06	0.95									
2. Opposition to free trade	2.90	1.18	0.29^***^								
3. Opposition to free trade agreements	3.26	1.32	0.22^***^	0.29^***^							
4. Perceived trade threat	3.71	0.99	0.28^***^	0.44^***^	0.52^***^						
5. Resistance to social change	2.69	0.83	0.09	0.18^***^	0.09	0.26^***^					
6. Acceptance of social inequality	2.61	0.87	−0.02	−0.04	−0.19^***^	−0.14^**^	−0.14^**^				
7. Populism	3.70	0.86	0.64^***^	0.26^***^	0.11^*^	0.25^***^	0.10^*^	−0.08			
8. Educational attainment	3.57	0.97	−0.16^**^	−0.16^**^	−0.07	−0.05	−0.02	0.03	−0.16^**^		
9. Task autonomy	3.03	0.93	−0.20^***^	−0.15^**^	−0.15^**^	−0.07	0.05	0.06	−0.18^***^	0.45^***^	
10. Household income	2.89	1.46	−0.22^***^	−0.12^*^	−0.07	−0.01	−0.02	0.12^*^	−0.19^***^	0.26^***^	0.24^***^

* *p < 0.05;*

** *p < 0.01;*

*** *p < 0.001; N = 391*.

A series of hierarchical (OLS) regression analyses were carried out to examine whether conspiracy mentality explains any additional variance in trade perceptions and attitudes, taking several covariates into account. Regressions diagnostics indicated no violations of model assumptions. Although several multivariate outliers were detected by means of Cook's distance (Cohen et al., 2003), excluding these cases does not substantially change the results. In the first step, generalized political attitudes, labor market skills and demographic characteristics were included in the regression model. In the second step, conspiracy mentality was added to the model. All continuous variables were converted to range from 0 to 1 to facilitate the interpretation of unstandardized regression coefficients^3^. The results are shown in Table 3.

**Table 3:** Hierarchical regression predicting anti-trade perceptions and attitudes.

	**Perceived trade** ** threat**		**Opposition to free** ** trade**		**Opposition to free trade agreements**	
**Predictor**	**B (SE)**	**Beta**	**B (SE)**	**Beta**	**B (SE)**	**Beta**
Step 1: Δ*R*^2^	0.144^***^		0.147^***^		0.096^***^	
Resistance to social change	0.18^***^	0.22	0.15^**^	0.16	0.07	0.06
	(0.04)		(0.05)		(0.05)	
Acceptance of social inequality	−0.07	−0.09	0.01	0.01	−0.17^***^	−0.17
	(0.04)		(0.04)		(0.05)	
Populism	0.07	0.10	0.11	0.12	−0.06	−0.06
	(0.05)		(0.06)		(0.07)	
Educational attainment	−0.01	−0.02	−0.09^*^	−0.11	−0.02	−0.02
	(0.04)		(0.04)		(0.05)	
Task autonomy	−0.02	−0.03	−0.02	−0.02	−0.07	−0.07
	(0.04)		(0.05)		(0.05)	
Household income	0.04	0.09	−0.01	−0.02	0.01	0.01
	(0.02)		(0.03)		(0.03)	
Age	−0.02	−0.03	−0.09^*^	−0.10	−0.10^*^	−0.10
	(0.04)		(0.04)		(0.05)	
Male	−0.03	−0.09	−0.05^**^	−0.13	−0.05^*^	−0.11
	(0.02)		(0.02)		(0.02)	
East Germany	0.03	0.08	0.03	0.06	0.04	0.07
	(0.02)		(0.02)		(0.03)	
Step 2: Δ*R*^2^	0.021^**^		0.015^**^		0.026^***^	
Conspiracy mentality	0.14^**^	0.19	0.14^**^	0.16	0.20^***^	0.22
	(0.04)		(0.05)		(0.06)	
Constant	0.30^***^		0.25^***^		0.46^***^	
	(0.05)		(0.05)		(0.06)	
Total *R*^2^	0.165^***^		0.162^***^		0.122^***^

*The entries are unstandardized OLS regression coefficients, standard errors in parentheses, and standardized coefficients in the final step. All continuous variables ranged from 0 to 1; N = 391*.

* *p < 0.05*,

** *p < 0.01*,

*** *p < 0.001*.

In the first step, generalized political attitudes, labor market skills, and demographic characteristics accounted for 14.4% of the variance in perceived trade threats, Δ*R*^2^ = 0.144, *F*_(9, 381)_ = 7.10, *p* < 0.001, 14.7% of the variance in trade hostility, Δ*R*^2^ = 0.147, *F*_(9, 381)_ = 7.32, *p* < 0.001, and 9.6% of the variance in opposition to trade agreements, Δ*R*^2^ = 0.096, *F*_(9, 381)_ = 4.48, *p* < 0.001. The *R*^2^ change statistic between Step 1 and Step 2 indicated that an additional 2.1% of variance in perceived threat was explained by adding conspiracy mentality to the model, Δ*R*^2^ = 0.021, *F*_(1, 380)_ = 9.65, *p* = 0.002. The incremental increases in explained variance for disapproval of foreign trade and trade agreements were 1.5% [Δ*R*^2^ = 0.015, *F*_(1, 380)_ = 6.81, *p* =0.009] and 2.6%, [Δ*R*^2^ = 0.026, *F*_(1, 380)_ = 11.44, *p* < 0.001], respectively.

Mirroring the findings of the correlation analysis, conspiracy mentality significantly predicted increased perceptions of threats associated with the global exchange of goods and services, heightened hostility toward free trade, and stronger opposition to trade agreements. As can be seen from the regression coefficients in Table 3, moving from the minimum to the maximum score for conspiracy mentality increases the perceived threats from trade and general trade hostility by 14 percentage points (*b* = 0.14, *SE* = 0.04, β = 0.19, *t* = 3.11, *p* = 0.002, and *b* = 0.14, *SE* = 0.05, β = 0.16, *t* = 2.61, *p* = 0.009, respectively). Regarding opposition to trade agreements, changing from the lowest to the highest level of conspiracy mentality leads to a shift of 20 percentage points of the range of the dependent variable (*b* = 0.20, *SE* = 0.06, β = 0.22, *t* = 3.38, *p* = 0.001).

None of the other explanatory factors showed a comparable consistent and substantial effect on anti-trade attitudes and beliefs in the final step of the regression analysis. With regard to the other predictors in the final model, only generalized political attitudes had considerable effects on protectionism. As shown in Table 3, there was again a significant positive relationship between resistance to change and the perceived threats of trade relations as well as general animosity toward trade. Acceptance of inequality was a significant negative predictor of trade agreements between the EU and other countries. After adjusting for other predictors in the model, populist attitudes and individual differences in labor market skills did not make a noteworthy contribution to predicting foreign trade attitudes.

## Discussion

Opponents of foreign trade from both the left and the right of the political spectrum often argue that global trade serves the economic interests of powerful corporate capitalism, which tries to control national authorities and to undermine the liberal and social rights of ordinary people (Spark, 2000). In the present study, I examined whether a generalized belief that our society is controlled by the activities of conspiratorial powers influences attitudes toward free trade. Although the magnitude of effects are in the low to medium range, the results show that conspiracy mentality predicts unique variance in opposition to trade openness, even controlling for several drivers of protectionist attitudes such as generalized political attitudes and individuals' labor market skills. Furthermore, individuals who are more prone to a conspiratorial worldview feel more threatened by the economic, political, and social consequences of international trade. The results also show that resistance to social change makes an important contribution to explaining economic protectionism, which is in line with previous studies that found strong negative effects of related constructs such as authoritarianism on trade openness. By contrast, acceptance of social inequality was negatively associated with animosity toward trade agreements. While populist attitudes were positively associated with protectionism in the bivariate analysis, they did not contribute to the explanation of protectionist sentiments after adjusting for general beliefs in conspiracies. This lends further support to the discriminant validity of conspiracy mentality. The results also confirm once again that material interests do not play a major role in explaining trade attitudes compared to intergroup attitudes.

From a theoretical point of view, the results imply that a new dimension of intergroup attitudes should be taken into account in the psychological explanation of foreign trade preferences. Previous studies suggest that people reject free trade because they perceive trade relations as a zero-sum game (ethnocentrism), associate the exchange of goods with a negative influence of foreign ideas on their own culture (authoritarianism), or are afraid of losing their own status in competition with supposedly inferior nations (social dominance orientation). However, thinking in conspiracy categories makes not inferior but powerful groups responsible for the negative effects of economic globalization. This makes conspiracy theories particularly attractive to powerless groups in society who see themselves on the economic losing side.

The results also have practical implications for policies addressing concerns about economic globalization. Thus far, labor market and social policy measures have mainly been designed to compensate the so-called “losers of globalization” only materially by expanding welfare services (see Marcal, 2001; Hays et al., 2005). However, the reported findings make it clear that political countermeasures to economic nationalism should also leverage economic education and debunking techniques (van der Linden and Roozenbeek, 2021) to help people better understand global economic processes and to avoid the pitfalls of conspiratorial thinking.

The strengths of the present study are that the analyses are based on a diverse sample and, unlike other studies, rely on a relatively large number of items to measure foreign trade perceptions and attitudes. In addition, the scale used to measure conspiracy mentality is relatively abstract and is not, like other scales, based on a selection of beliefs in specific conspiracy theories (e.g., Brotherton et al., 2013). Thus, the scale is not contaminated with political and economic positions that one might want to explain.

Naturally, however, the conclusions are also subject to some limitations. The present sample is restricted to German citizens with Internet access drawn from an opt-in online panel. While Internet penetration in Germany is quite high, future studies should strive to replicate the results using probability samples that include the off-line population as well. Another limitation is that the cross-sectional and observational design of the study does not allow for any statements on the causal relationship between conspiracy mentality and protectionism. There is evidence that experimental exposure to political conspiracy theories increases distrust in governmental institutions (Einstein and Glick, 2015; Kim and Cao, 2016), and it could be worthwhile to manipulate the salience of conspiratorial thinking to investigate the effects on economic attitudes and behavior. The reliabilities of the scales measuring resistance to change and acceptance of inequality were far from optimal, and future studies should strive to assess the incremental validity of conspiracy mentality using more established scales such as right-wing authoritarianism (Altemeyer, 1996). Likewise, future research could profit from including measures of nationalism to prove the robustness of the findings (e.g., Rankin, 2001). Moreover, it would be desirable to consider a more extensive number of covariates for material interests to account for the potentially confounding effects of the sector of employment, automation, or offshorability (e.g., Owen and Johnston, 2017).

## Conclusion

The evidence presented here contributes to the growing research on trade attitudes by showing that conspiracy mentality is an important factor in forming trade policy preferences. Economic globalization is associated not only with subjective fears of material losses but also with irrational ideas about being controlled by the invisible hand of dark forces. To be sure, not all criticisms of globalization are based on conspiratorial thinking, but people high in this trait are more likely to link trade issues with the supposed actions of sinister groups that pull the strings of international financial capitalism, thus creating a breeding ground for populist reactions to international trade.

## Data Availability Statement

The data that support the findings of this study are openly available from PsychArchives at: http://dx.doi.org/10.23668/psycharchives.4840.

## Ethics Statement

The studies involving human participants were reviewed and approved by the Ethics Committee at GESIS—Leibniz-Institute for the Social Sciences (Application 2020-2). The patients/participants provided their written informed consent to participate in this study.

## Author Contributions

The author confirms being the sole contributor of this work and has approved it for publication.

## Conflict of Interest

The author declares that the research was conducted in the absence of any commercial or financial relationships that could be construed as a potential conflict of interest.

## Footnotes

^1^The percentage of missing values was relatively low (≤ 6.2%), except for self-reported household income (10.2%) and attitudes toward trade agreements (11.2%). However, Little's (1988) test indicated that the data were missing completely at random, $\chi_{\left(148\right)}^{2}$ = 160.83, *p* = 0.22. Thus, listwise deletion was considered unproblematic. Nevertheless, missing values for household income were imputed using predictive mean matching to avoid losing too many observations. All variables from the analytical model were included in the imputation model as well as employment status as auxiliary variable. As suggested by the literature attitudes toward trade agreements as a dependent variable were not imputed (Little, 1992).

^2^An exception to this pattern is that conspiracy mentality is mainly associated with economic and symbolic threat (see Supplementary Table 3-1).

^3^The conversion was done using the following formula: (observed–min)/(max–min), with observed indicating the observed score for a given variable, and min and max denoting the minimum and maximum possible score on the scale, respectively (see Cohen et al., 2003).
