# Supplementary material for: Conspiracy Mentality Predicts Public Opposition to Foreign Trade
Source: Front Psychol. 2021 Jun 17;12:658919. doi: 10.3389/fpsyg.2021.658919 (PMC8247654; doi:10.3389/fpsyg.2021.658919)
Supplement: Supplementary file 1 [file Data_Sheet_1.pdf]

Supplementary Material

Conspiracy Mentality Predicts Public Opposition to Foreign Trade

https://dx.doi.org/10.3389/fpsyg.2021.658919

Table of Contents

1 Items and Question Wording

2 Demographic Composition of the Sample

3 Supplementary Analyses

1 Items and Question Wording

1.1 Conspiracy Mentality

Now we are going to ask you on your opinion on some general political issues. For each of the following statements, please indicate to what extent you agree or disagree with it.

- Most people do not recognize the extent to which our lives are determined by conspiracies that are concocted in secret.
- There are secret organizations that have a great influence on political decisions.
- Politicians and other leaders are nothing but the puppets of powers operating in the background.
- I think that the various conspiracy theories circulating in the media are absolute nonsense.*
- There is no good reason to distrust governments, intelligence agencies, or the media.*

Items marked with an asterisk are reverse coded. Response scale: strongly disagree (1), disagree (2), neither agree nor disagree (3), agree (4), strongly agree (5).

1.2 Attitudes toward International Trade

What do you think about the growing trade relations between Germany and other countries – do you think it is a good thing, or a bad thing for Germany?

Response scale: very bad (1), bad (2), somewhat bad (3), neither good nor bad (4), somewhat good (5), good (6), very good (7).

Currently, there is much discussion about the adoption of free trade agreements. Do you favor or oppose that the European Union (EU) enters free trade agreements with other countries?

Response scale: strongly favor (1), favor (2), somewhat favor (3), neutral (4), somewhat oppose (5), oppose (6), strongly oppose (7).

1.3 Perceived Threats Posed by International Trade

How do you see the effects of growing world trade on Germany: Does global trade have more positive or negative effects on the following areas?

- Economic growth
- Employment and labor market situation
- Consumer prices for goods and services
- International competitiveness
- Consumer protection standards (e.g., for agricultural products)
- Environmental standards
- Workers' rights and social standards
- Cultural life
- State regulatory sovereignty
- Our cultural values and traditions

Response scale: very positive (1), positive (2), somewhat positive (3), neutral (4), somewhat negative (5), negative (6), very negative (7).

1.4 Generalized Political Attitudes

1.4.1 Resistance to Social Change

- The world is constantly changing, and we should adapt our understanding of moral behavior to these changes. *
- We should be more tolerant of people who choose to live according to their own moral standards, even if they are very different from our own. *

1.4.2 Acceptance of Social Inequality

- Only if the differences in income and social standing are large enough will there be an incentive for personal performance.
- The differences in rank between people are acceptable because they essentially express what you did with the opportunities you had.
- I think the social differences in our country are generally fair.

Items marked with an asterisk are reverse coded. Response scale: strongly disagree (1), disagree (2), neither agree nor disagree (3), agree (4), strongly agree (5).

1.4.3 Populism

- The members of the German Bundestag should be committed exclusively to the will of the people.
- The people, and not politicians, should make the most important political decisions.
- The political differences between the elite and the people are greater than the differences among the people.
- An ordinary citizen would represent my interests better than a professional politician.
- Politicians talk too much and take too little action.
- What is called compromise in politics is really just selling out on one’s principles.

Items marked with an asterisk are reverse coded. Response scale: strongly disagree (1), disagree (2), neither agree nor disagree (3), agree (4), strongly agree (5).

1.5 Labor Market Skills

1.5.1 Education

What general school leaving certificate do you have?

Response scale: Still in school (1), lowest secondary qualification (Hauptschulabschluss), after 9 years of schooling (2), intermediary secondary qualification (Realschulabschluss), after 10 years of schooling (3), higher secondary qualification (Fachhochschulreife/Fachoberschule), after 11 or 12 years of schooling (technical college entrance qualification) (4), higher secondary qualification (Abitur), after 12 or 13 years of schooling (general higher education entrance qualification), without secondary school leaving certificate (6)

1.5.2 Vocational qualification

What vocational or professional training do you have?

Response scale: Still in vocational training (vocational preparation year, trainee, intern, student) (1), apprenticeship (2), vocational school (3), training at technical college, master craftsman's school, technical school, vocational or technical academy (4), University or technical college degree (Bachelor, Master, etc.) (5) Other vocational training qualification (open) (6), no completed vocational qualification, not in vocational training (7).

1.5.3 Monthly Household Income

If you take the income of all the members of your household together: What is the average monthly net income of all household members, that is, the sum of all income, including all benefits and social security payments, in total?

Response scale: less than EUR 500 (1), EUR 500 to less than EUR 1,000 (2), EUR 1,000 to less than EUR 1,500 (3), EUR 1.500 to less than EUR 2.000 (4), EUR 2.000 to less than EUR 2.500 (5), EUR 2.500 to less than EUR 3.000 (6), EUR 3.000 to less than EUR 3.500 (7), EUR 3.500 to less than EUR 4.000 (8), EUR 4.000 to less than EUR 4.500 (9), EUR 4.500 to less than EUR 5.000 (10), EUR 5.000 to less than EUR 10.000 (11), EUR 10.000 or more (12).

1.5.4 Occupational Status

(Filter: If worked full-time or part-time)

To which group does your current or former occupational activity belong?

- (1) Worker
- (2) Employee
- (3) Civil servant, judge, soldier
- (4) Academically independent profession (e.g., doctor with own practice, lawyer)
- (5) Self-employed individuals in trade, commerce, industry, services, etc.
- (6) Self-employed farmer
- (7) Assisting family member(s)
- (8) In vocational training
- (97) not applicable
- (99) no answer

Occupational Status, Worker

And what exactly applies to you?

- (1) unskilled and semiskilled worker
- (2) qualified and skilled worker
- (3) foreman

- (97) not applicable
- (99) no answer

Occupational Status, Employee

And what exactly applies to you?

- (1) Employee, with simple tasks (e.g., salesperson, clerk, shorthand typist)
- (2) Employee who performs difficult tasks independently according to general instructions (e.g., clerical assistant, accountant, technical draftsman)
- (3) Employee who performs independent work in a responsible position or who bears limited responsibility for the work of others (e.g., research assistant, authorized signatory, head of department, master craftsman in employment)
- (4) Employee with comprehensive management tasks and decision-making powers (e.g., director, managing director, board of directors of larger companies and associations)
- (97) not applicable
- (99) no answer

Occupational Status, Civil Servant

And what exactly applies to you?

- (1) lower grade of civil service or in a comparable category
- (2) middle grade of civil service or in a comparable category
- (3) upper grade of civil service or in a comparable category
- (4) higher grade of civil service or in a comparable category
- (97) not applicable
- (99) no answer

Occupational Status, Freelancer, Self-employed person

And what exactly applies to you?

- (1) without employees
- (2) 1-4 employees
- (3) 5 employees or more
- (97) not applicable
- (99) no answer

Occupational Status, Farmer

And what exactly applies to you?

- (1) with a utilized agricultural area of less than 10 hectares
- (2) with a utilized agricultural area of 10 hectares or more
- (3) Cooperative farmer
- (97) not applicable
- (99) no answer

Table 1-1. Index of Vocational Qualification (adapted from Hoffmeyer-Zlotnik & Warner 2005)

	**Formal Education**				
**Vocational qualification**	Still in school, without school leaving certificate	Lowest secondary qualification	Intermediary secondary qualification	Higher secondary qualification I	Higher secondary qualification II
Still in vocational training	1	1	1	2	2
Apprenticeship	3	3	3	4	4
Vocational school	3	3	3	4	4
Training at technical college, master craftsman's school, technical school, vocational or technical academy	4	4	4	4	4
University or technical college degree (Bachelor, Master, etc.)	x	4	4	5	5
No completed vocational qualification, not in vocational training	1	1	2	3	3

Table 1-2. Index of Occupation Status (adapted from Hoffmeyer-Zlotnik & Geis, 2003)

Index Score	Occupation
1	Unskilled and semiskilled worker
2	Qualified and skilled worker; Employee, with simple tasks (e.g., salesperson, clerk, shorthand typist); Lower grade of civil service or in a comparable category; Farmer with a utilized agricultural area of less than 10 hectares; Cooperative farmer
3	Foreman; Employee who performs difficult tasks independently according to general instructions (e.g., clerical assistant, accountant, technical draftsman); Middle grade of civil service or in a comparable category; Freelancer, Self-employed person without employees; Farmer with a utilized agricultural area of 10 hectares or more; Assisting family member(s)
4	Employee who performs independent work in a responsible position or who bears limited responsibility for the work of others (e.g., research assistant, authorized signatory, head of department, master craftsman in employment); Upper grade of civil service or in a comparable category; Freelancer, Self-employed person with 1-4 employees
5	Employee with comprehensive management tasks and decision-making powers (e.g., director, managing director, board of directors of larger companies and associations); Higher grade of civil service or in a comparable category; Freelancer, Self-employed person with 5 employees or more

2 Demographic Composition of the Sample

Table 2.1. Demographic Composition of the Sample Compared to Population Benchmarks

	Study Sample (n = 391)	*Population*	*Discrepancy (Sample – Population)*
**Gender**			
Male	51.7%	49.2%	2.5%
Female	48.1%	50.8%	-2.7%
Diverse	0.3%	-	0.3%
**Age**			
18-29	14.1%	18.9%	-4.8%
30-44	20.2%	22.2%	-2.0%
45-59	28.1%	26.8%	1.3%
60+	37.6%	32.1%	5.5%
**Education**			
Low (left school without degree or lower secondary qualification, after 9 years of schooling	20.7%	36.3%	-15.6%
Medium (intermediary secondary qualification, after 10 years of schooling	43.5%	30.0%	13.5%
High (higher secondary qualification, after 11+ years of schooling, technical college or university entrance qualification)	35.8%	33.5%	2.3%

Note. Percentages may not sum to 100 due to rounding. The population benchmarks are projections based on the 2019 Microcensus, an annual representative survey of one percent of individuals and households in Germany conducted by the Federal Statistical Office. The Microcensus uses a stratified cluster sampling methodology and, due to the obligation to provide information, the household response rate is about 94 percent.

3 Supplementary Analyses

Table 3-1. Hierarchical Regression Predicting Different Facets of Trade Threat

Predictor	Economic Threat		Threats to Consumer and Working Standards		Symbolic Threat	
	B (*SE*)	*Beta*	*B* (*SE*)	*Beta*	*B* (*SE*)	*Beta*
Step 1: ΔR^2	0.146***		0.114***		0.147***	
Resistance to Social Change	0.16***	0.18	0.17***	0.18	0.26***	0.28
	(0.04)		(0.05)		(0.04)	
Acceptance of Social Inequality	-0.02	-0.03	-0.16***	-0.18	-0.00	-0.00
	(0.04)		(0.04)		(0.04)	
Populism	0.09	0.10	0.05	0.06	0.09	0.10
	(0.05)		(0.06)		(0.06)	
Educational Attainment	-0.03	-0.04	0.02	0.03	-0.04	-0.05
	(0.04)		(0.04)		(0.04)	
Task Autonomy	-0.02	-0.02	-0.03	-0.03	0.00	0.00
	(0.04)		(0.05)		(0.05)	
Household Income	0.02	0.04	0.07*	0.13	0.02	0.04
	(0.03)		(0.03)		(0.03)	
Age	-0.04	-0.05	-0.01	-0.01	-0.03	-0.04
	(0.04)		(0.04)		(0.04)	
Male	-0.04*	-0.10	-0.03	-0.08	-0.00	-0.01
	(0.02)		(0.02)		(0.02)	
East Germany	0.04	0.08	0.03	0.06	0.04	0.08
	(0.02)		(0.02)		(0.02)	
Step 2: ΔR^2	0.034***		0.004		0.015*	
Conspiracy Mentality	0.19***	0.25	0.07	0.09	0.13*	0.16
	(0.05)		(0.05)		(0.05)	
Constant	0.22***		0.43***		0.21***	
	(0.05)		(0.05)		(0.05)	
Total R^2	0.180***		0.118***		0.163***	

Note: The entries are unstandardized OLS regression coefficients, standard errors in parentheses, and standardized coefficients in the final step. All continuous variables ranged from 0 to 1, $N = 389$.
* $p < .05$, ** $p < .01$, *** $p < .001$.
